# WAKWiN-Mini-Review: intraoperative Beatmung und postoperative pulmonale Komplikationen – aktuelle Evidenz und Konzepte zur Lungenprotektion

**DOI:** 10.1007/s00101-026-01687-x

**Published:** 2026-04-22

**Authors:** Katharina Hellenthal, Martin Scharffenberg

**Affiliations:** 1 Klinik für Anästhesiologie, operative Intensivmedizin und Schmerztherapie, Universitätsklinikum Münster, Münster, Deutschland; 2 Division of Pulmonary and Critical Care Medicine, Massachusetts General Hospital, Harvard Medical School, Boston, MA USA; 3 Klinik und Poliklinik für Anästhesiologie und Intensivtherapie, Pulmonary Engineering Group, Universitätsklinikum Carl Gustav Carus, Technische Universität Dresden, Fetscherstraße 74, 01307 Dresden, Deutschland

**Keywords:** Beatmungs-assoziierte Lungenschädigung, Tidalvolumen, PEEP, Driving Pressure, Mechanische Beatmungsleistung, Ventilator-induced lung injury, Tidal volume, PEEP, Driving pressure, Mechanical power

## Abstract

Etwa 5 % der Patienten erleiden nach chirurgischen Eingriffen in Allgemeinanästhesie postoperative pulmonale Komplikationen. In 20 % dieser Fälle versterben die Patienten innerhalb der ersten 30 postoperativen Tage. Die maschinelle Beatmung stellt den pulmonalen Gasaustausch während der Allgemeinanästhesie sicher, kann jedoch selbst zu einer beatmungsassoziierten Lungenschädigung führen. In Analogie zur Beatmung im akuten Lungenversagen wurden verschiedene intraoperative Beatmungsstrategien entwickelt, um postoperative pulmonale Komplikationen zu vermeiden. Während sowohl bei Ein- wie Zweilungenbeatmung bislang weder durch einen hohen positiven end-exspiratorischen Druck oder eine Individualisierung des *Driving Pressure *eine konsistente Reduktion postoperativer pulmonaler Komplikationen nachgewiesen werden konnte, deutet sich bei ausgewählten Eingriffen und Risikopatienten jedoch ein positiver Effekt an. Neuere pathophysiologische Konzepte wie die mechanische Beatmungsleistung (Mechanical Power bzw. Intensity) könnten zukünftig helfen, Beatmung zu optimieren und Komplikationen zu reduzieren. Diese Übersichtsarbeit bietet eine kompakte Zusammenfassung der Pathogenese einer beatmungsassoziierten Lungenschädigung und diskutiert die neueste verfügbare Evidenz sowie das Potenzial aktueller Beatmungskonzepte zur Lungenprotektion.

## Abstract (en)

Approximately 5% of patients undergoing surgery under general anesthesia suffer from postoperative pulmonary complications. In 20% of these cases, the patients die within the first 30 days after surgery. While ensuring pulmonary gas exchange under general anesthesia, intraoperative mechanical ventilation itself may substantially contribute to the development of ventilator-induced lung injury. Based on approaches used in the treatment of acute respiratory distress syndrome, various intraoperative ventilation strategies have been developed to prevent postoperative pulmonary complications. While neither a high positive end-expiratory pressure nor an individualization of the driving pressure has so far been shown to consistently reduce postoperative pulmonary complications in either one-lung or two-lung ventilation, a beneficial effect appears to emerge in selected surgical procedures and high-risk patients. Newer pathophysiological concepts such as mechanical power (or intensity) may help to optimize ventilation and reduce complications in the future. This review article provides a concise summary of the pathogenesis of ventilator-induced lung injury and discusses the most recently available evidence as well as the potential of current ventilation strategies for lung protection.

## Hintergrund

Etwa 5 % aller Patienten erleiden nach einer Allgemeinanästhesie postoperative pulmonale Komplikationen (PPC). Hierzu zählen Atelektasen, Pneumonien, das akute Lungenversagen (ARDS) sowie Aspirationen [2], wobei die Definition dieses zusammengesetzten Endpunktes nicht immer einheitlich ist. In etwa 20 % ziehen diese Komplikationen innerhalb der ersten 30 postoperativen Tage den Tod des Patienten nach sich. Bei weltweit über 310 Mio. Operationen jährlich entspricht dies einer signifikanten Anzahl Betroffener [28]. Neben patienten- und eingriffsspezifischen Risikofaktoren beeinflusst die intraoperative Beatmung das Outcome maßgeblich.

In der Pathogenese der beatmungsassoziierten Lungenschädigung spielen insbesondere bei prolongierter Beatmung Barotrauma sowie Volutrauma, die durch die Erhöhung des transpulmonalen Drucks (Stress) und Dehnung der Lunge (Strain) das Lungenparenchym schädigen, eine entscheidende Rolle. Andererseits führt ein zyklisches Kollabieren und Wiedereröffnen der Alveolen zum sog. Atelektrauma. Die dabei vermittelte Mechanotransduktion kann als Biotrauma zusammengefasst werden [17]. Diese Stimuli schädigen die alveolokapilläre Einheit, induzieren Immunzellrekrutierung und Ödemausbildung [23], während die Schädigung des Alveolarepithels die Surfactant-Synthese und damit Compliance einschränkt [18, 40].

Das Risiko für eine Lungenschädigung und PPC ist bei einer Einlungenventilation (ELV) noch höher (ca. 30 %). Während der ELV steht weniger Lungenparchenchym für Beatmung und Gasaustausch zur Verfügung. Die ventilierte Lunge befindet sich in Seitenlage meist unten und wird dabei durch Schwerkrafteffekte und einen ggf. zusätzlich angelegten Kapnothorax komprimiert. Die respiratorische Mechanik ist hierdurch mitunter maßgeblich beeinträchtigt. Die erhaltene Perfusion der nichtventilierten Lunge bedingt einen funktionellen Rechts-links-Shunt. Während der ELV ist das Risiko zwischen Hypoventilation/Hypoxämie gegenüber einem erhöhten Beatmungsdruck individuell abzuwägen. Auch extrapulmonale Komplikationen können aufgrund von Zytokin-Distanz-Effekten ausgelöst werden [20]. Die nachfolgenden Beatmungsparameter bzw. -konzepte sollen dies verhindern.

## Tidalvolumen

Das Konzept der lungenprotektiven Beatmung hat seinen Ursprung in der ARDS-Therapie, bei der die Begrenzung des Tidalvolumens (V_T_) auf ≤ 6 ml/kg des idealen Körpergewichtes (PBW) die Letalität senkte [3]. Intraoperativ ist die Datenlage weniger eindeutig: Zwar reduzierte ein V_T_ ≤ 7 gegenüber > 10 ml/kg PBW in einer Cochrane-Analyse postoperative Pneumonien und Beatmungspflichtigkeit, jedoch nicht die Sterblichkeit [16]. In einer randomisiert-kontrollierten Studie mit 1236 Patienten unterschied sich die PPC-Inzidenz nicht zwischen V_T_ 6 und 10 ml/kg PBW [22]. Demzufolge tolerieren gesunde Lungen – im Gegensatz zum ARDS – offenbar zumindest temporär V_T_ > 6 ml/kg PBW, wobei V_T_ > 10 ml/kg PBW vermieden werden sollen [48]. Eine Metaanalyse zeigte allerdings kürzlich, dass das V_T_ generell Einfluss auf das PPC-Risiko haben kann [7]. Ein pragmatischer Ansatz ist die Anwendung von Zielwerten um 6–8 ml/kg PBW (Einlungenventilation: 4–6 ml/kg PBW); ein direkter Letalitätseffekt wird in zukünftigen Studien unter Beweis zu stellen sein [35, 42].

## Positiver end-exspiratorischer Druck

In großen Studien an normalgewichtigen und adipösen Patienten konnte ein erhöhter positiver end-exspiratorischer Druck (PEEP) mit Lungenrekrutierungsmanövern (12 vs. ≤ 2 bzw. 4 cm H_2_O) zwar eine Verbesserung von Lungenmechanik und Oxygenierung bewirken, aber ohne Reduktion der PPC und zulasten der Hämodynamik [53, 56]. Ähnliches konnte kürzlich in der randomisierten kontrollierten PROTHOR-Studie auch für die Einlungenbeatmung gezeigt werden, wo ein höherer PEEP in Kombination mit Rekrutierungsmanövern (10 vs. 5 cm H_2_O) ebenfalls zu keiner Reduktion der PPC führte [1]. Sekundäranalysen weisen jedoch auf potenzielle Vorteile höherer PEEP-Werte bei laparo-/thorakoskopischen Eingriffen und Risikopatienten hin [27].

## Driving Pressure

Der *Driving Pressure* (∆P), definiert als Differenz von Plateaudruck und PEEP, ist ein einfach ablesbarer Beatmungsparameter und gilt als Surrogat der Compliance. Die klinische Bedeutung des ∆P verdeutlichte eine retrospektive ARDS-Studie, bei der ein ∆P > 15 cm H_2_O mit einer erhöhten Sterblichkeit assoziiert war [5]. Eine individuelle PEEP-Titration mit Lungenrekrutierungsmanövern kann den ∆P, insbesondere bei adipösen Patienten oder laparoskopischen Eingriffen, gezielt reduzieren [8, 10, 31, 38, 51]. In der klinischen Praxis kann hierfür beispielsweise eine dekrementelle PEEP-Titration angewendet werden, wobei der PEEP ausgewählt wird, bei dem der ∆P am niedrigsten (bzw. die Compliance am höchsten) ist [15, 44]. Diese Individualisierung ermöglicht zwar eine optimierte Lungenmechanik, pulmonale Belüftung und verbesserten Gasaustausch [24, 25, 29, 54], führte aber häufig zu intraoperativen Hypotonien und erhöhtem Vasopressorbedarf [43, 53]. Die Evidenz zu direkten Effekten auf das PPC-Risiko ist außerdem widersprüchlich – nicht zuletzt aufgrund verhältnismäßig niedriger Fallzahlen (*n* = 46–694) [11, 12, 26]. In zwei großen Metaanalysen (17 bzw. 16 Studien) konnte schließlich eine signifikante Assoziation zwischen erhöhtem intraoperativem ∆P und dem Risiko für PPC nachgewiesen werden (OR = 0,358, *p* = 0,002; OR = 1,16, *p* < 0,001) [7, 32]. Allerdings führte eine ∆P-optimierte Beatmungsstrategie mit Rekrutierungsmanövern bei offener Abdominalchirurgie in der kürzlich publizierten randomisierten kontrollierten DESIGNATION-Studie jedoch zu keiner signifikanten Reduktion der PPC [52]. Die Ergebnisse der GENERATOR-Studie (NCT06101511) [47], die derzeit eine individualisierte Beatmung mit PEEP-Titration bei minimalinvasiver Abdominalchirurgie hinsichtlich PPC untersucht, gilt es, mit Spannung abzuwarten. Die Optimierung mittels PEEP-Titration wurde auch für die Einlungenventilation untersucht. Eine Studie zeigte zwar bereits eine reduzierte PPC-Rate bei ∆P 9 vs. 10 cm H_2_O (*n* = 292; OR = 0,42, *p* = 0,047) [33], diese Ergebnisse wurden allerdings hauptsächlich von Komplikationen in der nichtventilierten Lunge getragen und als statistisch schwach kritisiert [4]. In der adäquat gepowerten Folgestudie (*n* = 1170) fand sich neben verbesserter Compliance und Oxygenierung kein Unterschied im PPC-Auftreten (42,8 vs. 40,5 %, *p* = 0,42) [34]. Ebenso zeigte eine aktuelle Metaanalyse mit 13 RCT (*n* = 3401) keinen Vorteil einer ∆P-Optimierung [14]. In der klinischen Praxis kann die Titration des ∆P in Ein- und Zweilungenbeatmung helfen, Lungenmechanik und Oxygenierung zu verbessern, nicht aber PPC zu reduzieren. Laut aktueller Empfehlungen sollte während der Zweilungenventilation ein ∆P ≤ 14 cm H_2_O angestrebt werden [55], auch wenn dieser Richtwert primär aus der ARDS-Beatmung stammt.

## Mechanische Beatmungsleistung und Intensität

**Abb. 1:**
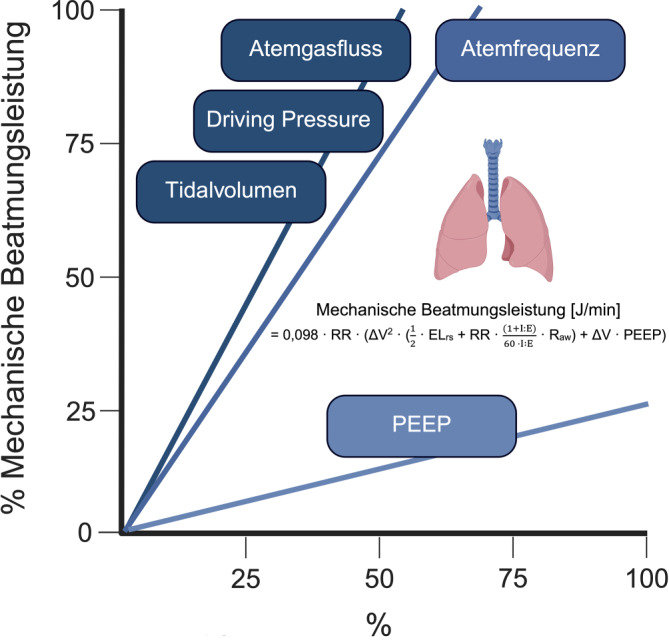
Theoretischer Einfluss verschiedener Einzelfaktoren auf die Beatmungsleistung, wobei der tatsächliche Einfluss einzelner Parameter noch kontrovers diskutiert wird [19]. Die Abbildung wurde von Gattinoni et al. adaptiert [13] und mit BioRender.com erstellt

Die Beatmungsleistung (*Mechanical Power*) beschreibt die durch Beatmung im respiratorischen System temporär gespeicherte und abgegebene mechanische Energie pro Zeit [9]. Sie fasst hierbei alle wesentlichen Beatmungsparameter zusammen, die potenziell auch allein eine beatmungsinduzierte Lungenschädigung verursachen können (Abb. 1).

In die Berechnung der Mechanical Power fließt insbesondere die Atemfrequenz ein. Hier konnte kürzlich gezeigt werden, dass eine Erhöhung der Atemfrequenz zur Reduktion des V_T_ mit einem erhöhten Letalitätsrisiko, vermittelt über eine Zunahme der Mechanical Power, assoziiert war [38, 49]. Neben den zuvor aufgeführten Beatmungsparametern wird auch der Atemgasfluss bei der Mechanical Power berücksichtigt, dem die flusskontrollierte Beatmung als neuere Beatmungsmodalität begegnen soll [41, 50].

Experimentell wurde ein direkter Zusammenhang der Mechanical Power mit einer Lungenschädigung nachgewiesen [39]. Klinisch zeigte sich ein Zusammenhang mit einer erhöhten postoperativen Reintubationsrate < 7 Tage: Ein Anstieg um 5 J/min erhöhte das Risiko um 31 % (*n* = 230.767, *p* < 0,001) [36]. Bei der Einlungenventilation zeigte das Reintubationsrisiko einen U‑förmigen Verlauf mit minimalem Risiko bei 6,4 J/min (*n* = 878) [45, 46]. Da eine ungeplante Reintubation bei postoperativer respiratorischer Insuffizienz mit einer 18fachen Steigerung des Letalitätsrisikos einhergeht [30], könnte die *Mechanical Power* auch die Letalitätsrate beeinflussen. Allerdings sind noch einige methodologische Limitationen zu beachten: Unterschiedliche Berechnungsformeln korrelieren bereits im experimentellen Setting nicht einheitlich mit Lungenschäden [37], zudem ist derzeit keine Routinebestimmung möglich, und die Exspiration bleibt mathematisch unberücksichtigt [19].

Die Beatmungsintensität (*Intensity*) normiert die Beatmungsleistung auf die funktionell verfügbare Lungenoberfläche, was die Vergleichbarkeit erleichtern könnte. Hierfür könnte idealerweise das endexspiratorische Lungenvolumen aus Computertomographie/Magnetresonanztomographie- oder Gasmessverfahren bzw. als intraoperativ verfügbare Surrogate Body Mass Index und Compliance herangezogen werden. So konnte kürzlich ein erhöhtes Risiko für PPC (OR = 1,34, *p* < 0,001) und akutes respiratorisches Versagen (OR = 1,4, *p* < 0,001) bei Erhöhung der Compliance-normierten *Mechanical Power* als *Intensity*-Surrogat gezeigt werden [21]. Protokolle für eine *Mechanical Power*-optimierte Beatmung und deren wissenschaftliche Evaluation stehen derzeit aus.

## Patienten- und eingriffsspezifische Faktoren

Der in randomisiert-kontrollierten Studien häufig ausgebliebene Effekt intraoperativer Beatmungsinterventionen auf das PPC-Risiko kann damit zusammenhängen, dass die intraoperative Beatmungsdauer im Vergleich zur prolongierten Beatmung auf der Intensivstation deutlich kürzer und damit der klinische Einfluss möglicherweise begrenzt ist. Nichtsdestotrotz konnte die klinische Outcome-Relevanz der intraoperativen Beatmung in großen Metaanalysen gezeigt werden [7, 15, 27, 32], weshalb diese nicht weniger sorgfältig durchgeführt werden sollte. Es muss aber auch angemerkt werden, dass der zusammengesetzte Endpunkt PPC nicht einheitlich definiert ist [2]. Insbesondere bestimmte Risikopatienten, wie Patienten mit kardiopulmonalen Vorerkrankungen, Adipositas, Raucher und ältere Patienten können womöglich von individualisierter, protektiver Beatmung profitieren. Beispielsweise werden Patienten mit Adipositas häufig mit per se zu hohen V_T_ beatmet [6]. Spontan atmende adipöse Patienten kompensieren hingegen die erhöhte Atemarbeit infolge ihres Übergewichts eher durch erhöhte Atemfrequenzen. Tidalvolumina sollten hierbei also streng auf das Idealkörpergewicht normiert werden. In der multizentrischen, randomisierten PROBESE-Studie zur Reduktion von PPC bei adipösen Patienten wurden diese entweder mit einem PEEP von 4 cm H_2_O oder PEEP von 12 cm H_2_O mit stündlichen Rekrutierungsmanövern beatmet. In der Gruppe des höheren PEEP konnten der Driving Pressure und intraoperative Hypoxien zwar deutlich reduziert werden, es war allerdings auch ein höherer Bedarf an Vasopressoren erforderlich, und ein signifikanter Effekt auf die PPC-Rate blieb letztendlich aus [53]. Neben den patientenspezifischen Risikofaktoren spielen intraoperative Bedingungen, wie Lagerung, Einlungenventilation, Operationsdauer oder Invasivität eine Schlüsselrolle. So wird beispielsweise bei der Etablierung eines Kapnoperitoneums die Compliance des Thorax durch kraniale Verlagerung des Zwerchfells reduziert, das endexspiratorische Lungenvolumen verringert, und es kommt zu einem Anstieg des arteriellen CO_2_ durch transperitoneale Resorption, dem mit einer Anpassung der Beatmung begegnet werden muss. Lungenprotektion erfordert daher bei bestimmten Risikokonstellationen eine patienten- und eingriffsspezifische Anpassung sowie perioperative statt reiner intraoperativer Ansätze.

## Fazit für die Praxis

- PPC stellen ein relevantes Problem dar und müssen reduziert werden.
- Bei der Zweilungenventilation sollten Tidalvolumina von 6–8 ml/kg PBW anvisiert werden und 10 ml/kg PBW keinesfalls überschreiten. Für die Einlungenventilation ist das V_T_ zu reduzieren (~5 ml/kg PBW).
- Ein hoher PEEP mit Rekrutierungsmanövern bei Ein- und Zweilungenventilation sollte kritisch betrachtet und abgewogen werden.
- Ein hoher PEEP oder eine *Driving-Pressure*-orientierte PEEP-Titration können während Ein- und Zweilungenbeatmung Lungenmechanik und Oxygenierung verbessern, scheinen PPC aber nicht zu reduzieren. Allerdings profitieren möglicherweise thorako-/laparoskopisch operierte Patienten von einem höheren PEEP.
- *Mechanical Power* und ihre Normierung als *Intensity* sind vielversprechende Konzepte, erfordern aber noch Weiterentwicklung und technische Implementierung.
- Grundlagenforschung, Translation und adäquat große randomisiert-kontrollierte Studien sowie Identifikation von Risikogruppen, Beatmungsindividualisierung und perioperative Ansätze könnten künftig entscheidend dazu beitragen, PPC zu reduzieren.

## Data Availability

Die für diese Übersichtsarbeit genutzte Literatur kann auf Anfrage von den Autoren zur Verfügung gestellt werden.
